# Stromal vapors for real-time molecular guidance of breast-conserving surgery

**DOI:** 10.1038/s41598-020-77102-1

**Published:** 2020-11-18

**Authors:** Pierre-Maxence Vaysse, Loes F. S. Kooreman, Sanne M. E. Engelen, Bernd Kremer, Steven W. M. Olde Damink, Ron M. A. Heeren, Marjolein L. Smidt, Tiffany Porta Siegel

**Affiliations:** 1 Division of Imaging Mass Spectrometry, Maastricht MultiModal Molecular Imaging Institute (M4I), University of Maastricht, Maastricht, The Netherlands; 2 Department of Surgery, Maastricht University Medical Center+, Maastricht, The Netherlands; 3 Department of Otorhinolaryngology, Head and Neck Surgery, Maastricht University Medical Center+, Maastricht, The Netherlands; 4 Department of Pathology, Maastricht University Medical Center+, Maastricht, The Netherlands; 5 GROW School for Oncology and Developmental Biology, Maastricht University Medical Center+, Maastricht, The Netherlands; 6 Department of General, Visceral and Transplantation Surgery, RWTH University Hospital Aachen, Aachen, Germany; 7 NUTRIM School of Nutrition and Translational Research in Metabolism Faculty of Health, University of Maastricht, Maastricht, The Netherlands

**Keywords:** Breast cancer, Surgical oncology, Mass spectrometry, Medical and clinical diagnostics

## Abstract

Achieving radical tumor resection while preserving disease-free tissue during breast-conserving surgery (BCS) remains a challenge. Here, mass spectrometry technologies were used to discriminate stromal tissues reported to be altered surrounding breast tumors, and build tissue classifiers ex vivo. Additionally, we employed the approach for in vivo and real-time classification of breast pathology based on electrosurgical vapors. Breast-resected samples were obtained from patients undergoing surgery at MUMC+. The specimens were subsequently sampled ex vivo to generate electrosurgical vapors analyzed by rapid evaporative ionization mass spectrometry (REIMS). Tissues were processed for histopathology to assign tissue components to the mass spectral profiles. We collected a total of 689 ex vivo REIMS profiles from 72 patients which were analyzed using multivariate statistical analysis (principal component analysis-linear discriminant analysis). These profiles were classified as adipose, stromal and tumor tissues with 92.3% accuracy with a leave-one patient-out cross-validation. Tissue recognition using this ex vivo-built REIMS classification model was subsequently tested in vivo on electrosurgical vapors. Stromal and adipose tissues were classified during one BCS. Complementary ex vivo analyses were performed by REIMS and by desorption electrospray ionization mass spectrometry (DESI-MS) to study the potential of breast stroma to guide BCS. Tumor border stroma (TBS) and remote tumor stroma (RTS) were classified by REIMS and DESI-MS with 86.4% and 87.8% accuracy, respectively. We demonstrate the potential of stromal molecular alterations surrounding breast tumors to guide BCS in real-time using REIMS analysis of electrosurgical vapors.

## Introduction

Breast conserving surgery (BCS) is a broadly used surgical treatment for early-stage breast cancer patients and consists of achieving full tumour removal while conserving as much as possible healthy tissues. Despite assistance options with intraoperative diagnostic techniques such as ultrasound or radioactive seed localization^1^, BCS can remain a challenge for the surgeon. In the current setting, the outcome of the operation can only be determined in detail after pathology examination of the resected tissue. An unsuccessful outcome can lead to reoperation or more burdensome adjuvant treatments for the patient^2^.

Despite its soft structure, recent advances in ambient mass spectrometry have enabled the analysis of breast tissues^3–6^ by desorption electrospray ionization mass spectrometry imaging (DESI-MSI)^7^ and rapid evaporative ionization mass spectrometry (REIMS)^8,9^ to predict histopathology based on metabolic profiles. While DESI-MSI uses charged droplets to desorb molecules from tissue sections to generate in situ two dimensional molecular distributions with precise histopathology examination, REIMS analyses electrosurgical vapors of tissue slices ex vivo and in vivo during surgery. After building a library of histologically validated lipid profiles with REIMS, tissues can be classified within seconds, which matches with the intraoperative need for pathological feedback^5,8,9^. As REIMS is easily combined to routine surgical tools, it may greatly benefit surgeons by providing an on-line feedback about tissue pathology based on the chemical information present in the vapors produced in vivo without changing the operation procedure. This is a crucial point to facilitate clinical implementation of the technology for in vivo metabolic profiling.

DESI has determined specific metabolic profiles for adipose, stromal, glandular and tumor of breast tissues^3,4^ but REIMS has discriminated normal breast and breast tumor tissues only^6^. The variety of healthy breast tissues reported with DESI-MSI suggests that specific metabolic profiles for different healthy breast tissues could be expected with REIMS, which remains unexploited. Moreover, alterations of stromal tissues surrounding breast tumors have been associated to clinical observations^10,11^. If detectable in real-time, these stromal changes could mark a safe area of resection to complement the assessment of margin of resection by histopathology. They would constitute a classification of healthy and tumor surrounding tissues for precise surgical guidance.

Herein, we investigate the potential of breast stroma to guide BCS by mass spectrometry analysis. First, we built a classification model based on REIMS analysis of ex vivo electrosurgical vapors to recognize breast stroma during BCS. Then, we use REIMS and DESI-MS to investigate further the potential of breast stroma to guide BCS.

## Materials and methods

### Study population and tissue procurement

This study included 85 female patients (Table S1) who underwent surgery for breast tumor at Maastricht University Medical Centre (MUMC+) between September 2017 and September 2019. The study patient inclusion followed a protocol approved by the Medical Ethics Committee (Medisch-Ethische ToetsingsCommissie) azM/UM of MUMC+ (approval number METC 16-4-168). The study was conducted with highest practice standards according to the revised version of the Declaration of Helsinki. Written informed consent was obtained from each patient prior to study participation. A pathologist selected samples on the resected-tissue for the present study; samples were taken of the tumor and benign tissue at least 2 cm away from the tumor. Tissue slices were used for REIMS ex vivo analysis, or frozen in liquid nitrogen and stored at − 80 °C until DESI-MSI analysis.

### REIMS ex vivo analysis

Tissues from 72 patients were cauterized ex vivo using a monopolar hand-piece (iKnife disposable device, Waters, Hungary) equipped with a 1.7 cm-diameter blade electrode, connected to an electrosurgical heat-generator (Force FX, Covidien), operated in cut modality. The generated vapors were aspirated into a mobile REIMS Xevo G2-XS Q-ToF mass analyzer (Waters Corporation, Wilmslow, UK). Isopropanol (Biosolv, France or Honeywell, Germany) containing Leucine-Encephalin (Sigma-Aldrich, The Netherlands) was infused at 150 μL/min^12^. REIMS acquisitions were performed in negative ionization mode over the mass range *m*/*z* 100 to 1500. After REIMS analysis, the remaining tissue was formalin fixed and paraffin embedded. Tissue blocks were sectioned at 5 μm-thickness. Tissue sections were stained with hematoxylin and eosin (H&E). A breast pathologist attributed tissue components to the surroundings of the sampling spots without knowledge of the MS profiles. Percentages of tissue components were assigned to each MS profile based on the histology surrounding of each sampling spot. For the classification of tumor border stroma (TBS) and remote tumor stroma (RTS), only the stroma profiles correctly classified in the adipose/tumor/stroma REIMS model were included. Stromal profiles generated on tissue sampled at least 2 cm from the tumor and presenting no tumor were considered RTS profiles. Stroma profiles generated on tissues containing sampling spots containing a tumor component in their pathology examination were considered TBS profiles.

### REIMS in vivo analysis

In vivo measurement was performed on the same instrument during the BCS of one patient. Surgery was performed using commercial hand-piece (Erbe) and heat generator (Valleylab FT10, Covidien). Three GoPros (Hero4) were set up to record surgical site, heat generator and screen of the computer of the mass spectrometer to coordinate in time, site of electrosurgical vapors production, the diathermia parameters and MS profile generation. Synchronization was performed using timestamps associated to sound generated in the operation room during the surgery. Orientation of the resection margins on the specimen was obtained during and after surgery using video recorded and comments made by surgeons at that time and while watching the video post-surgery. Macroscopic and microscopic analyses of the resection margins were inspected by a pathologist according to the surgeons’ markings. Conditions for experimental measurements were set up the same as for ex vivo analysis with air gas. Electrosurgical vapors were directed towards the mass spectrometer and the REIMS source and partially discarded as usual.

### DESI-MS analysis

Two groups of specimens were selected for DESI-MSI analysis; samples of the tumor site or samples of fibro-glandular tissues at least 2 cm away from the tumor site from 22 patients. Frozen tissues were sectioned using a cryotome (Microm) at 10 μm-thickness, thaw mounted on histological slides (Superfrost) and stored at − 80 °C prior analysis. Experiments were performed on a Xevo G2-XS Q-ToF MS (Waters Corporation, Wilmslow, UK). 98%methanol/water (Biosolv, France) at 1.5 μl/min was used as a solvent. Measurements used for the tissue classification were performed in negative ionization mode, over the mass range *m*/*z* 100–1000 at 40 × 40 μm^2^ pixel size, acquired at scan rate of 150 μm/s. Tissue sections were stained by H&E and scanned on a slide scanner (Ventana) after DESI-MSI analysis. Stromal areas surrounding remote normal glands and surrounding tumors were selected by a breast pathologist using QuPath (v0.1.2). One data point corresponded to the sum of the signal of 6 adjacent pixels selected in TBS or RTS areas in HD Imaging (v1.5, Waters, UK). Complementary experiment was performed over the mass range *m*/*z* 50–2000 at 30 × 30 μm^2^ pixel size at scan rate of 100 μm/s.

### Data analysis

Data were analyzed using a prototype of abstract model builder software (AMX v1.01563.0, Waters Research Corporation, Budapest, Hungary). One scan was considered per tissue sampling spot. Data processing includes lock-mass correction, background subtraction, normalization on total ion count and signal background removal. Principal component analysis-linear discriminant analysis (PCA-LDA) and leave-one patient-out cross-validation were used to compare the accuracy between the histopathology classification and the MS-based classification.

### Molecular identification

Spectra were lock-mass corrected on deprotonated leucine-encephalin *m*/*z* 554.2615 [M–H]^−^ for REIMS and deprotonated raffinose *m*/*z* 503.1606 [M–H]^−^ for DESI-MS for identification based on mass accuracy. Tandem mass spectrometry experiment was performed by collision-induced dissociation with argon gas. Experimental data were tested on ALEX^123^ lipid calculator^13^ for fatty acids and compared to reference literature for lactate dimer^4^.

### Statement

We report the in vivo recognition of breast stroma and its potential for real-time molecular guidance of breast-conserving surgery using rapid evaporative ionization mass spectrometry analysis of electrosurgical vapors.

## Results

### Ex vivo built database enables in vivo tissue recognition

In total, 689 REIMS ex vivo profiles were generated from 72 patients and were attributed as 209 stroma, 256 adipose and 224 tumor profiles by histopathology (Tables S2–3).

**Figure 1:**
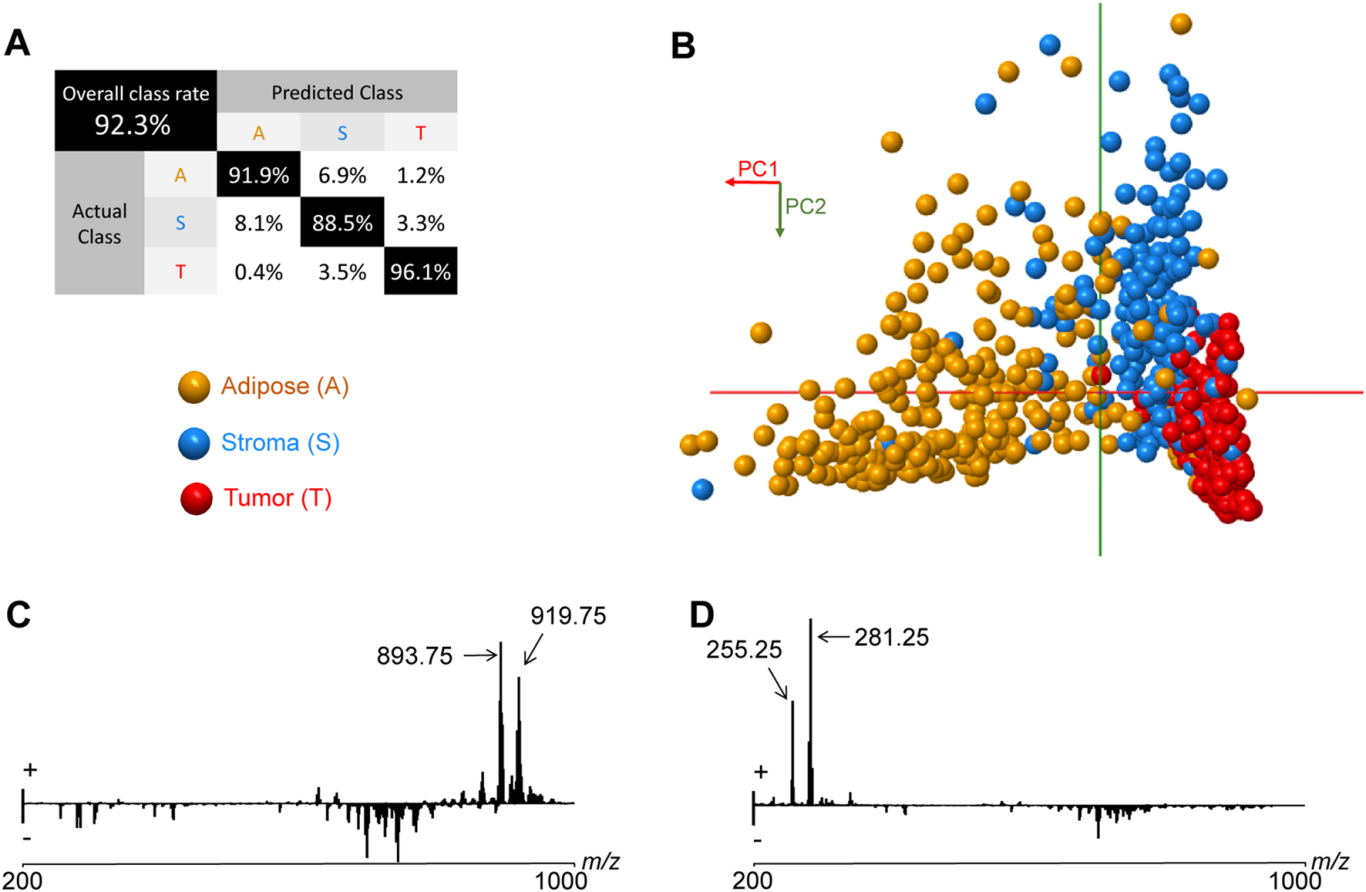
REIMS analysis of electrosurgical vapors ex vivo classifies tumor, stroma and adipose tissues. (**A**) PCA score plot (mass range *m*/*z* 200–1000, PC1 describing 56.2% of total variance, PC2 25.6%). (**B**) Confusion matrix. (**C**) Mass features loading plots for PC1 with indication of the two most discriminative mass features for adipose. (**D**) mass features loading plots for PC2 with indication of the two most discriminative mass features for stroma.

REIMS profiles were classified with 92.6% accuracy (Fig. 1A) using PCA-LDA and a leave-one patient-out cross-validation. A PCA score plot (Fig. 1B) showed a separation of adipose and tumor profiles along the PC1 axis (which describes 56.2% of the variance of the data) and a separation of stroma and tumor profiles along the PC2 axis (25.6%). Main discriminators were *m*/*z* 893.75 and *m*/*z* 919.75 (Fig. 1C) previously assigned as triglycerides^6^ for adipose, and *m*/*z* 255.25 and *m*/*z* 281.25 (Fig. 1D) assigned as fatty acids, palmitic acid and oleic acid respectively, for stroma (Table S5). Single MS profiles for each tissue type and pseudo-LDA score plot are shown in Figure S1.

**Figure 2:**
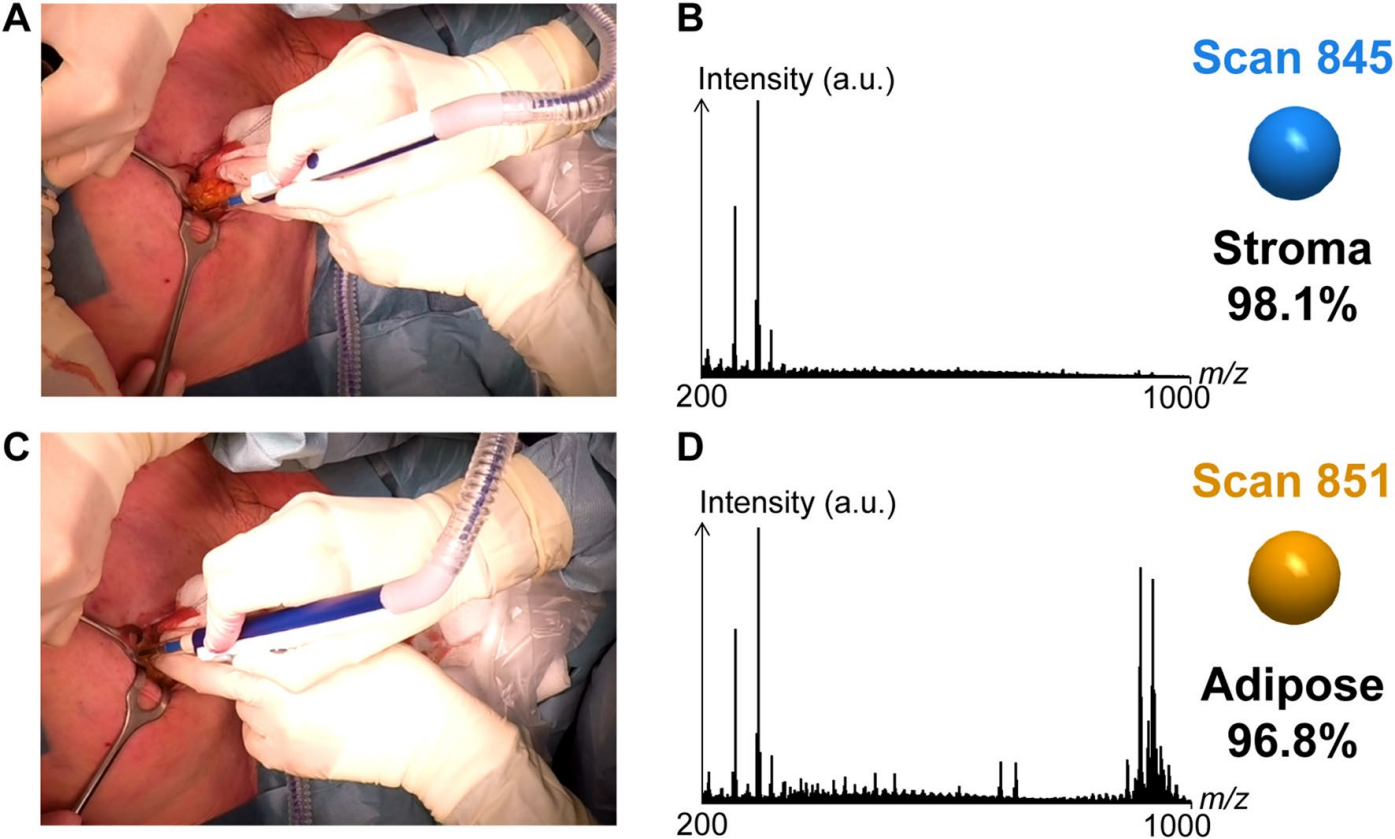
REIMS analysis of electrosurgical vapors enables in vivo tissue recognition. (**A**) Surgical site for scan 845. (**B**) Mass spectrum signal and tissue classification result for scan 845. (**C**) Surgical site for scan 851. (**D**) Mass spectrum signal and tissue classification result for scan 851.

Next, in vivo REIMS data were collected in an operating room during one BCS performed using a diathermia knife. We detected intense MS signals throughout the operation (Fig. S2A). At around 14 min of recording, scan 845 and scan 851 (Fig. 2B, D, respectively), both displayed MS signals generated in cut modality. The pictures taken during surgery indicate a change in the transected tissue (Fig. 2A, C). While the most intense MS peaks in scan 845 were detected only in the mass range *m*/*z* 200–400, intense MS peaks were displayed in the mass range *m*/*z* 800–1000 for scan 851.

Our REIMS model of ex vivo profiles enabled the recognition of stroma on scan 845 and of adipose on scan 851. Based on pathology report, stroma and adipose tissues were present on the resection margins present on the mediodorsal orientation of the specimen (Fig. S2B and S2C respectively). These observations correlated with the MS signal measured in scans 845 and 851. This demonstrates the potential of our REIMS classification model generated ex vivo to recognize breast tissue types in vivo during surgery.

### Stroma changes surrounding breast tumors

**Figure 3:**
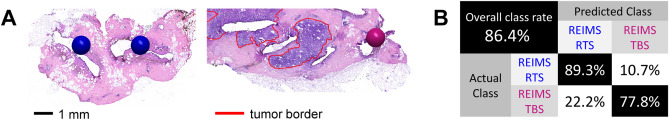
REIMS profiles discriminate tumor border stroma (TBS) and tumor remote stroma (TRS). (**A**) Histological sampling spots examined as RTS and TBS. (**B**) Confusion matrix (mass range *m*/*z* 200–500).

Further investigations were pursued on 189 ex vivo REIMS profiles generated from tissues of 43 patients and designed as 44 tumor border stroma (TBS) profiles and as 140 remote tumor stroma (RTS) profiles (Table S3; Fig. 3A). PCA score plot did not enable separation of TBS and RTS profiles, therefore no straightforward screening and molecular identification of the main MS peaks discriminant of TBS and RTS was possible (Fig. S3A). However, LDA score plot enabled a good separation (Fig. S3B) and MS profiles were classified as TBS or RTS with 86.5% accuracy (Fig. 3B) using PCA-LDA and a leave-one patient-out cross-validation.

**Figure 4:**
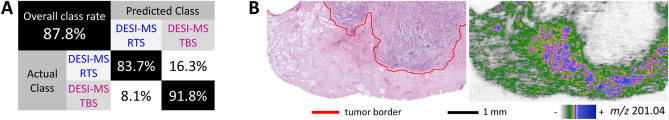
DESI-MS profiles discriminate tumor border stroma (TBS) and tumor remote stroma (TRS). (**A**) Confusion matrix (mass range *m*/*z* 200–400). (**B**) Histology and molecular distribution of lactate dimer (*m*/*z* 201.04) in a histologically normal stroma surrounding an invasive ductal carcinoma.

We complemented the REIMS experiments with DESI-MSI experiments for more precise analysis of the stromal changes surrounding tumor borders. In total, 196 DESI-MS profiles, including 98 remote tumor stroma profiles (RTS) and 98 tumor border stroma (TBS) profiles, were extracted from pixels of 22 tissue sections from 22 patients (Table S4). DESI-MS profiles were classified as TBS or RTS with 87.8% accuracy (Fig. 4A) using PCA-LDA and leave-one patient-out cross-validation. PCA score plot displayed a separation RTS and TBS mainly along the PC1 axis (Fig. S4A). Main discriminator of TBS along PC1 mass loading plot was a mass feature *m*/*z* 201.05 (Fig. S4C) corresponding to mass value *m*/*z* 201.04 previously identified (15) and confirmed (Table S6) as lactate dimer. Its distribution delineates the tumor borders with an intense MS signal coming from the TBS areas (Fig. 4B), illustrating how in situ molecular distributions may provide relevant information beyond histopathology.

## Discussion

We confirm the changes of metabolic profiles between remote and tumor stromas on intraoperative breast biopsies by DESI-MSI^4^. We report similar classification of stromal tissues with REIMS analysis of electrosurgical vapors. Our ex vivo built library of histopathology validated molecular profiles enabled in vivo stroma recognition and therefore illustrates a exciting step towards intraoperative molecular guidance during BCS. Metabolic alterations of the tumor microenvironment lay the bases of how insight into molecular changes can improve surgical precision.

Technological improvements of speed and intraoperative sampling^14–16^ recently contributed to emerging mass spectrometric tissue classifiers for clinical applications^17^. Paradoxically, the molecular margin of resection^18^ beyond histopathology, characterized in earlier studies to understand tumor recurrence^19,20^, was set aside as a factor to be considered in the most recent investigations. The benefit of mass spectrometric technologies for clinical applications has been mainly considered only by histopathological validation which remains the gold standard for disease-free patient survival. Nevertheless, the detection of more subtle molecular changes, not detectable by histopathology, could be even more valuable, as previously reported by REIMS on primary liver tumors^8^. REIMS has been mainly reported in the perspective to correct the execution of unsafe resection margins by near real-time classification of tumor and normal tissues during surgery. Our report suggests that surgeons could benefit from a critical information, the distance to the tumor, with recognition of breast stroma metabolic profiles during surgery. Beyond a correction, this would constitute a real-time molecular guidance to improve surgical precision of BCS. This is expected to be a major leap towards achieving the smallest possible resections with safe margins. Our classification of breast pathology from electrosurgical vapors generated ex vivo enabled direct stroma recognition in vivo. This augments the value of REIMS for in vivo tissue recognition^6^ and to utilize breast stromal molecular information to guide more precise BCS.

## Supplementary information

Supplementary information.

Supplementary Figure Legends.

Supplementary Figure 1.

Supplementary Figure 3.

Supplementary Figure 4.

## Data Availability

The process datasets generated during the current study are available with this article in the supporting material, accessible from *Scientific reports* website.
