# Supplementary material for: Stromal vapors for real-time molecular guidance of breast-conserving surgery
Source: Sci Rep. 2020 Nov 18;10:20109. doi: 10.1038/s41598-020-77102-1 (PMC7674429; doi:10.1038/s41598-020-77102-1)
Supplement: Supplementary file 1 — Supplementary information. [file 41598_2020_77102_MOESM1_ESM.pdf]

Stromal vapors for real-time molecular guidance of breast-conserving surgery
supplementary files

Pierre-Maxence Vaysse,[1,2,3] Loes F. S. Kooreman,[4,5] Sanne M. E. Engelen,[2,5] Bernd Kremer,[3,5] Steven W. M. Olde Damink,[2,6,7] Ron M. A. Heeren,[1] Marjolein L. Smidt,[2,5] and Tiffany Porta Siegel[1]

[1] Maastricht MultiModal Molecular Imaging Institute (M4I), Division of Imaging Mass Spectrometry, University of Maastricht, The Netherlands

[2] Department of Surgery, Maastricht University Medical Center+, The Netherlands

[3] Department of Otorhinolaryngology, Head & Neck Surgery, Maastricht University Medical Center+, The Netherlands

[4] Department of Pathology, Maastricht University Medical Center+, The Netherlands

[5] GROW School for Oncology and Developmental Biology, Maastricht University Medical Center+, The Netherlands

[6] Department of General, Visceral and Transplantation Surgery, RWTH University Hospital Aachen, Aachen, Germany.

[7] NUTRIM School of Nutrition and Translational Research in Metabolism Faculty of Health, University of Maastricht, The Netherlands

Table S1. Baseline characteristics of the patient population. IQR: interquartile range, BMI: body mass index

Studies	All combined	DESI-MS	REIMS *ex vivo*	REIMS *in vivo**
Number of patients	85	22	72	1
Clinical characteristics I				
Age, years median, IQR	61 (53-72)	57 (53-72)	61 (53-72)	69
BMI, kg/m^2 median, IQR	25.8 (23.7-29.2)	25.6 (24.3-28.8)	26.2 (24.1-29.4)	23.1
Patients with unknown BMI	9 (11)	3	9	0
Clinical characteristics II, number of patients (%)				
Parity				
P0	13 (15%)	3 (14%)	11 (15%)	
P1	9 (11%)	4 (18%)	7 (10%)	
P2	35 (41%)	10 (45%)	29 (40%)	
P3	15 (18%)	3 (14%)	13 (18%)	1
P4 or more	3 (4%)	1 (5%)	2 (3%)	
Unknown	10 (12%)	1 (5%)	10 (14%)	
Tabaco use				
Active or recent sevrage	18 (21%)	4 (18%)	16 (22%)	
Past or absent	61 (72%)	17 (77%)	52 (72%)	
Unknown	6 (7%)	1 (5%)	5 (7%)	
Pathological characteristics, number of patients (%)				
Tumor stage				
Tis	4 (5%)	3 (14%)	2 (3%)	
T0	3 (4%)	0 (0%)	3 (4%)	
T1	50 (59%)	13 (59%)	42 (58%)	1
T2	25 (29%)	5 (23%)	23 (32%)	
T3/4	3 (4%)	1 (5%)	2 (3%)	
Tumor grade				
Not applicable	5 (6)	3 (14%)	3 (4%)	
Grade I	8 (9)	2 (9%)	7 (10%)	1
Grade II	41 (48)	10 (45%)	33 (46%)	
Grade III	31 (36)	7 (32%)	29 (40%)	
Lymph node status				
Nx	1 (1%)	0 (0%)	1 (1%)	
N0	55 (65%)	15 (68%)	46 (64%)	1
N1	22 (26%)	6 (27%)	18 (25)	
N2/3	9 (11%)	1 (5%)	7 (10%)	
Treatment, number of patients (%)				
Surgery				
Breast-conserving surgery	28 (33%)	3 (14%)	25 (35%)	1
Ablation	57 (67%)	19 (86%)	47 (65%)	
Neoadjuvant therapy				
Yes	12 (14%)	3 (14%)	10 (14%)	
No	73 (86%)	19 (86%)	62 (86%)	1

*The results reported for the *in vivo* REIMS experiments were performed during the surgery of a patient operated for a single invasive ductal carcinoma of no special type, grade 2, estrogen receptor +, progesterone receptor +, epidermal growth factor receptor 2-.

Table S2. Pathological characteristics of the tumor profiles included in the library for the REIMS *ex vivo* study. N.A.: unknown or not applicable, DCIS: ductal carcinoma *in situ*, IDC: invasive ductal carcinoma, IDLC: invasive ducto-lobular carcinoma, ILC: invasive lobular carcinoma, ER: estrogen receptor, PR: progesterone receptor: HER2: human epidermal growth factor receptor 2

	Tumor type				ER/PR status				HER2 status			Tumor grade			
	DCIS	IDC	IDLC	ILC	ER+/PR+	ER+/PR-	ER-/PR-	N.A.	HER2+	HER2-	N.A.	I	II	III	N.A.
Profiles	3	176	1	44	158	30	28	8	46	171	7	9	76	136	3
Patients	2	35	1	12	34	8	5	3	6	41	3	3	21	24	2

Table S3. Number of REIMS profiles per tissue type and per patient included in the library for the REIMS *ex vivo* study. P: patient, A: adipose, T: tumor, S: stroma, RTS: remote tumor stroma, TBS: tumor border stroma

REIMS	A	T	S	RTS	TBS	TOTAL	REIMS	A	T	S	RTS	TBS	TOTAL
P1	1	8				9	P37	1	6	5	4		12
P2	2	8	5	5		15	P38	2	1				3
P3	2	5	2		1	9	P39		4	3	3		7
P4	6	2				8	P40			5	3	2	5
P5	5	2	4	2	2	11	P41	1	16				17
P6	2	2	2		2	6	P42	2	4	14	13		20
P7		1	4		4	5	P43		1				1
P8	2	7	4		4	13	P44			10	10		10
P9		9	11	10	1	20	P45	1					1
P10	2	16	7		7	25	P46	6		2	2		8
P11		7				7	P47	3		3	3		6
P12	6	4	5	4		15	P48	3	1				4
P13	3	1				4	P49	16	1	6	4		23
P14	1	1				2	P50	5	3	10	9	1	18
P15	10					10	P51	22	5	1		1	28
P16	3	7	1			11	P52	14		4	1		18
P17		6	5	4	1	11	P53	7		1		1	8
P18	1		6	6		7	P54	1	6				7
P19		5	6	3	3	11	P55	1	2	5	5		8
P20	2	5				7	P56	13	5				18
P21	2	2				4	P57	5		2	1		7
P22	6	15				21	P58	5		1			6
P23		4	5	5		9	P59	4	7	2	1		13
P24	1		8	4	3	9	P60			4		3	4
P25	4	3	1		1	8	P61	13	1				14
P26	2		6	4		8	P62	3	2	10	9		15
P27	1	2				3	P63	9	2	5	2	3	16
P28			7	7		7	P64	3	3	3	3		9
P29	10	2				12	P65	12					12
P30	2		6	6		8	P66	4					4
P31	2	9	1			12	P67	4	5	1			10
P32		4				4	P68	3	2				5
P33		1	3	2	1	4	P69	6					6
P34		3				3	P70	3		4	2	1	7
P35			1		1	1	P71	3	5	1		1	9
P36			6	3		6	P72	3	1	1		1	5
							TOTAL	256	224	209	140	45	689

Table S4. Number of REIMS profiles per tissue type and per patient included in the library for the DESI-MS study to classify tumor border stroma (TBS) and remote tumor stroma (RTS). P: patient.

DESI-MS	RTS	TBS	TOTAL
P74	9		9
P75	9		9
P10*		6	6
P12*	5		5
P76		9	9
P17*	9		9
P30*	12		12
P31*		8	8
P33*		9	9
P77	9		9
P78		10	10
P49*		9	9
P79	9		9
P55*	9		9
P80		8	8
P62*		9	9
P64*	9		9
P81		9	9
P82		9	9
P83		12	12
P84	9		9
P85	9		9
TOTAL	98	98	196

*patient also provided tissues for the REIMS analysis.

Table S5. Lipid identification by REIMS based on mass accuracy.

Identification	Adduct	Formula	PCA *m/z*	Measured *m/z*	Theoretical *m/z*	Mass error (ppm)
NEFA 16:0	$-H^+$	$C_{16}H_{31}O_2$	255.25	255.23	255.23	1.4
NEFA 18:0	$-H^+$	$C_{18}H_{35}O_2$	281.25	281.25	283.26	0.7

Table S6. Lactate dimer identification by DESI-MS based on mass accuracy and tandem MS.

Identification	Adduct	Formula	PCA *m/z*	Measured *m/z*	Theoretical *m/z*	Mass error (ppm)	Fragment ion upon MS/MS
Lactate dimer	$[2M+Na]^-$	$C_6H_{10}O_6Na$	201.05	201.04	201.04	4	89.0

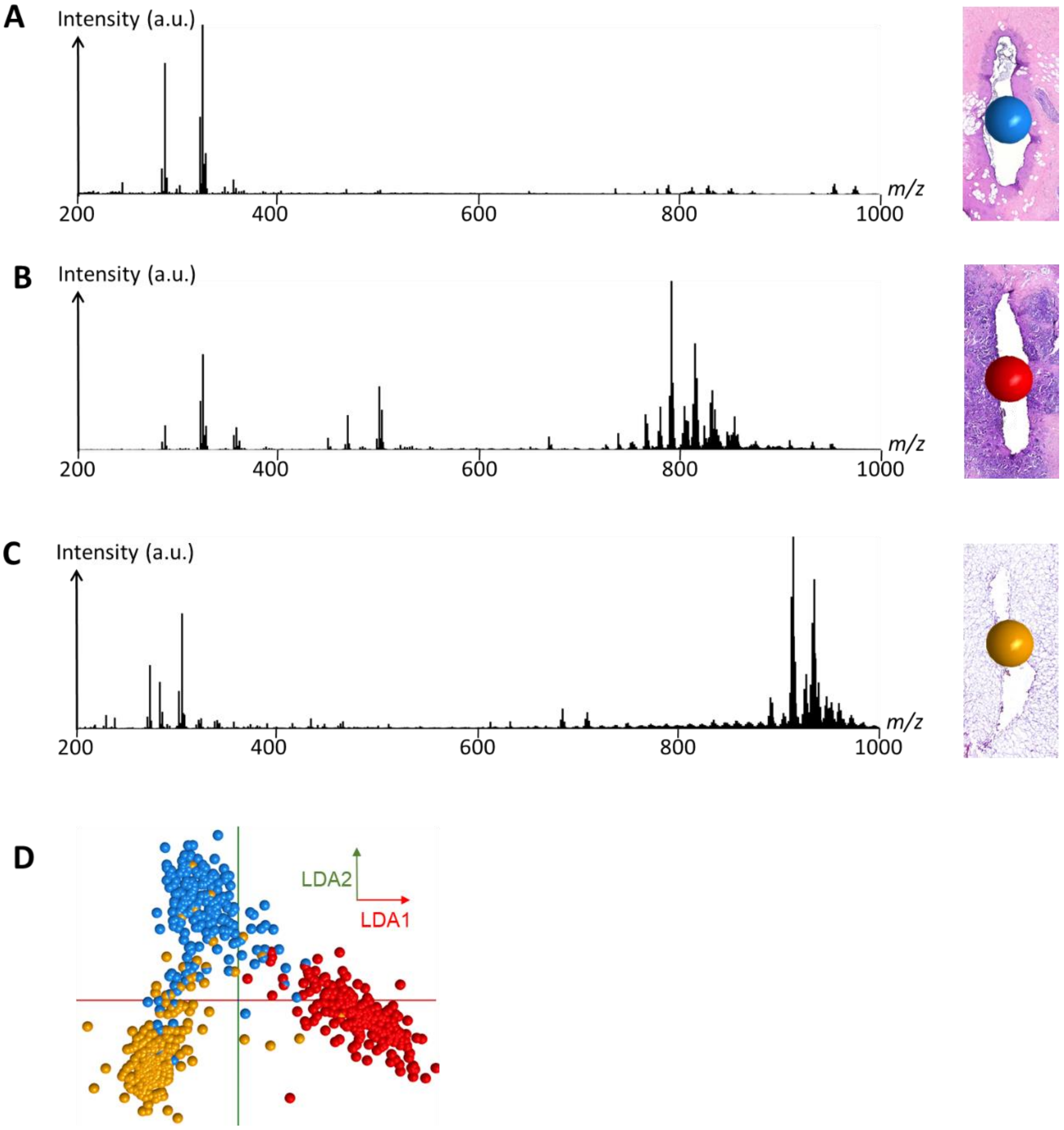

Figure S1. REIMS analysis of electrosurgical vapors *ex vivo* classifies tumor, stroma and adipose tissues.
A, mass spectral profile generated from stromal tissue with corresponding histology surrounding the sampling spot.
B, mass spectral profile generated from adipose tissue with corresponding histology surrounding the sampling spot.
C, mass spectral profile generated from tumor tissue with corresponding histology surrounding the sampling spot.
D, Pseudo-LDA score plot (mass range *m/z* 200-1000).

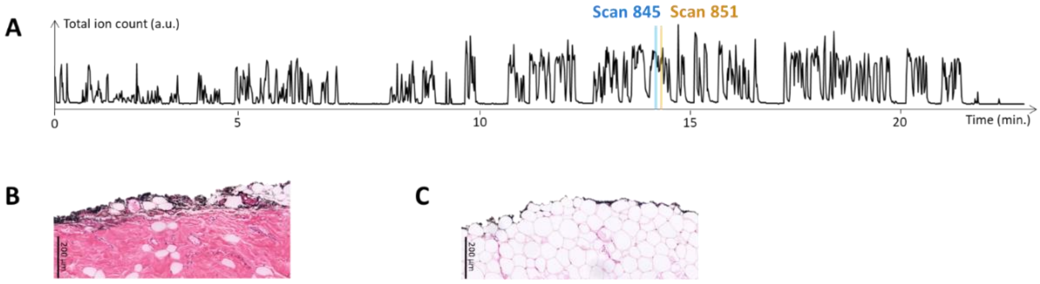

Figure S2. REIMS analysis of electrosurgical vapors enables *in vivo* tissue recognition during a BCS.
A, total ion count as a function of time analyzed by REIMS of electrosurgical vapors *in vivo* during a BCS.
B, probable histology of the resection margin based on the specimen orientation for scan 845, comporting substantial proportion of stroma tissue.
C, probable histology resection margin based on the specimen orientation for scan 851, comporting substantial proportion of adipose tissue.

.

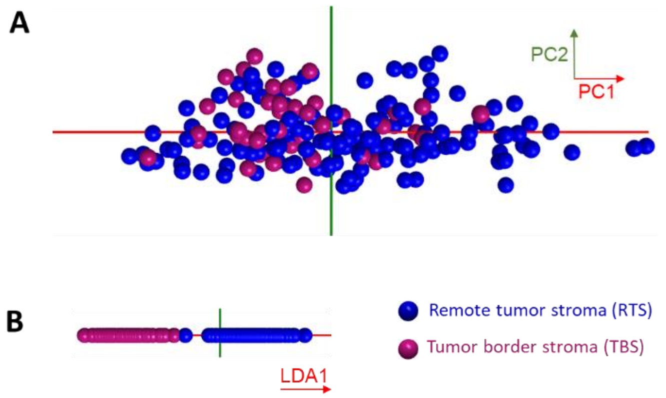

Figure S3. REIMS profiles discriminate tumor border stroma (TBS) and tumor remote stroma (TRS).
A, PCA score plot (mass range *m/z* 200-500, PC1 describing 82.6% of total variance, PC2 7.3%).
B, LDA score plot.

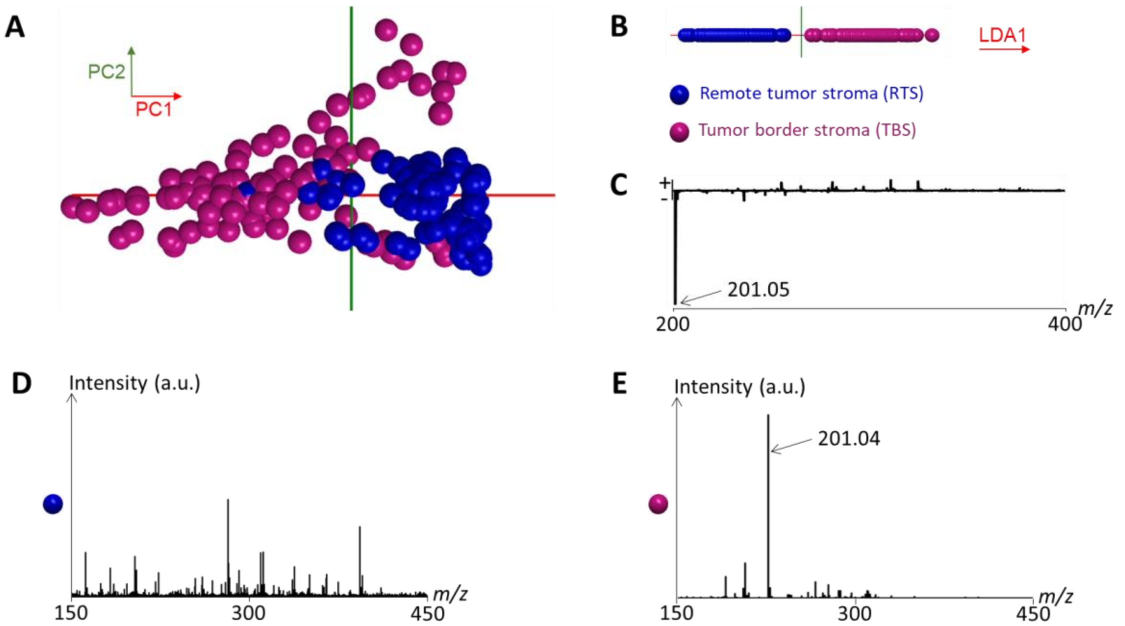

Figure S4. DESI-MS profiles discriminate tumor border stroma (TBS) and tumor remote stroma (TRS).
A, PCA score plot (mass range *m/z* 200-400, PC1 describing 76.8% of total variance, PC2 12.6%).
B, LDA score plot.
C, PC1 loading plot with indication of the most discriminative mass feature for TBS.
D, single DESI mass spectrum of TRS.
E, single DESI mass spectrum of TBS.
