# Supplementary material for: Stromal vapors for real-time molecular guidance of breast-conserving surgery
Source: Sci Rep. 2020 Nov 18;10:20109. doi: 10.1038/s41598-020-77102-1 (PMC7674429; doi:10.1038/s41598-020-77102-1)
Supplement: Supplementary file 2 — Supplementary Figure Legends. [file 41598_2020_77102_MOESM2_ESM.docx]

**Stromal vapors for real-time molecular guidance of breast-conserving surgery**

Pierre-Maxence Vaysse,^1,2,3^ Loes F. S. Kooreman,^4,5^ Sanne M. E. Engelen,^2,5^ Bernd Kremer,^3,5^ Steven W. M. Olde Damink,^2,6,7^ Ron M. A. Heeren,^1^ Marjolein L. Smidt,^2,5^ and Tiffany Porta Siegel^1^

^1^ Maastricht MultiModal Molecular Imaging Institute (M4I), Division of Imaging Mass Spectrometry, University of Maastricht, The Netherlands

^2^ Department of Surgery, Maastricht University Medical Center+, The Netherlands

^3^ Department of Otorhinolaryngology, Head & Neck Surgery, Maastricht University Medical Center+, The Netherlands

^4^ Department of Pathology, Maastricht University Medical Center+, The Netherlands

^5^ GROW School for Oncology and Developmental Biology, Maastricht University Medical Center+, The Netherlands

^6^ Department of General, Visceral and Transplantation Surgery, RWTH University Hospital Aachen, Aachen, Germany.

^7^ NUTRIM School of Nutrition and Translational Research in Metabolism Faculty of Health, University of Maastricht, The Netherlands

**Legends for the three supplementary Excel databases in the supplementary file:**

**File: Processed_data_Figure-1_MAM_REIMS_A_S_T.data.xlsx**
Processed data file for REIMS analysis of electrosurgical vapors ex vivo classifies tumor, stroma and adipose tissues. The data matrix was generated by abstract model builder software (AMX v1.01563.0, Waters Research Corporation, Budapest, Hungary).
In column A: assigned tissue classes (T: tumor; S: stroma; A: adipose tissue). In column B: anonymized patient identification number. In raw 1: mass channels (binning 0.10).

**File: Processed_data_Figure-3_MAM_REIMS_TBS_RTS.data.xlsx**
Processed data file for REIMS profiles discriminate tumor border stroma (TBS) and tumor remote stroma (TRS). The data matrix was generated by abstract model builder software (AMX v1.01563.0, Waters Research Corporation, Budapest, Hungary).
In column A: assigned tissue classes: tumor border stroma (TBS) and tumor remote stroma (TRS). In column B: anonymized patient identification number. In raw 1: mass channels (binning 0.10).

**File: Processed_data_Figure-4_MAM_DESI_TBS_RTS.data.xlsx**
Processed data file for DESI-MS profiles discriminate tumor border stroma (TBS) and tumor remote stroma (TRS). The data matrix was generated by abstract model builder software (AMX v1.01563.0, Waters Research Corporation, Budapest, Hungary).
In column A: assigned tissue classes: tumor border stroma (TBS) and tumor remote stroma (TRS). In column B: anonymized patient identification number. In raw 1: mass channels (binning 0.10).
